# Influence of ABO blood group on susceptibility to different pathological types of lung cancer: a retrospective study

**DOI:** 10.1186/s12957-022-02845-2

**Published:** 2022-12-04

**Authors:** Haotian Yang, Xianjun Zeng, Yu Zhang, Weilai Tong, Geliang Yao, Chunyu Lan, Jiaming Liu, Zhili Liu, Nanshan Zhong

**Affiliations:** 1 Medical Innovation Center, The First Affiliated Hospital of Nanchang University, Jiangxi 330006 Nanchang, People’s Republic of China; 2 Medical School of Nanchang University, Jiangxi 330031 Nanchang, People’s Republic of China; 3 Department of Information, The First Affiliated Hospital of Nanchang University, Jiangxi 330006 Nanchang, People’s Republic of China; 4 Institute of Spine and Spinal Cord, Nanchang University, Jiangxi 330006 Nanchang, People’s Republic of China

**Keywords:** ABO blood group, Lung cancer, Different pathological types, Logistic regression

## Abstract

**Purpose:**

Current research has shown a link between ABO blood group and many diseases. The purpose of this study aimed to investigate the influence of the ABO blood group on the risk of developing different pathological types of lung cancer.

**Materials and methods:**

This retrospective study was composed of 7681 patients with lung cancer and 12, 671 non-lung cancer patients who were admitted to the First Affiliated Hospital of Nanchang University from January 2016 to January 2021. The subjects with lung cancer were grouped into small cell lung cancer group (*n* = 725), lung adenocarcinoma group (*n* = 4520), and lung squamous cell carcinoma group (*n* = 2286) according to pathological types. The ABO blood group distribution of each lung cancer type group was compared with that of the control group. Statistical analysis was determined with chi-square and logistic regression.

**Results:**

Univariate analysis showed that the ABO blood group distribution of lung adenocarcinoma, lung squamous cell carcinoma, and small cell lung cancer was different from that of the control group (*P* < 0.01). After adjusting for age, sex, smoking history, and drinking history, logistic regression analysis showed that the risk of lung adenocarcinoma in blood type O was higher than that in blood type A (*P* < 0.01). There was no significant difference in ABO blood group composition between small cell lung cancer group, lung squamous cell carcinoma group, and control group (*P* > 0.05). In addition, gender and age have an influence on all three types of lung cancer (*P* < 0.01). Smoking was a risk factor in lung squamous cell carcinoma and small cell carcinoma (*P* < 0.01). Alcohol consumption was a risk factor in lung adenocarcinoma (*P* < 0.01).

**Conclusion:**

ABO blood group may be correlated with the occurrence of lung adenocarcinoma in Jiangxi province, but not with lung squamous cell carcinoma and small cell carcinoma.

## Introduction

Lung cancer is one of the cancers with the highest morbidity and mortality worldwide [1]. According to Global Cancer Statistics in 2020, lung cancer is the leading cause of morbidity and mortality among all malignancies in men. Among women, lung cancer is third in incidence and second in mortality [1]. By 2020, the incidence of lung cancer accounted for 11.4% of the total cancer population, and its mortality accounted for 18.0% of the total cancer population [1]. The epidemiological study of lung cancer is conducive to the evaluation and prediction of lung cancer to formulate the prevention and treatment plan. Histologically, lung cancer is divided into non-small cell lung cancer (NSCLC) and small cell lung cancer (SCLC), accounting for 85% and 15%, respectively [2]. Non-small cell lung cancer is divided into lung adenocarcinoma, squamous cell lung carcinoma, and large cell lung cancer [3]. Adenocarcinoma is the most common type of lung cancer, accounting for half of all lung cancer cases [4]. Epidemiological investigation of lung cancer is an important measure to prevent and cure the occurrence and development of lung cancer. It is also the key to reducing mortality and improving lung cancer prognosis.

ABO blood groups, discovered by Landsteiner in the last century, were the first polymorphic genetic phenotypes to be revealed. Due to its stable heritability and correlation with diseases, it has been highly valued by medical researchers. In recent years, more and more reports have been made on the relationship between the ABO blood group and malignant tumors. The difference between the ABO blood group and the occurrence and development of various cancers has a specific correlation, such as breast cancer, stomach cancer, pancreatic cancer, kidney cancer, and so on [5]. However, there were few reports on the relationship between blood type and the occurrence and prognosis of lung cancer, and there were some conflicting conclusions. In 2007, a large cohort of 3346 men enrolled in a 16-year Danish study showed that men with blood type O were more likely to die from lung cancer due to inflammatory factors [6]. In contrast, Unal et al. found no correlation between the ABO blood group and the prognosis of patients with advanced lung cancer in a study of 81 cases [7]. In 2015, a retrospective analysis of the Cancer Prevention and Treatment Center of Sun Yat-sen University suggested that blood type O and blood type B were favorable prognostic factors for the overall survival of NSCLC [8].

Different histopathological types of lung cancer have different etiology and pathogenesis. The purpose of this study is to explore the correlation between ABO blood group and various types of lung cancer. This will provide new ideas for the prediction, diagnosis, and treatment of lung cancer.

## Material and methods

### Patient characteristics

This study was a retrospective observational study. The newly treated lung cancer patients admitted to the Department of Respiratory Medicine of the First Affiliated Hospital of Nanchang University from January 2016 to January 2021 were selected by inclusion and exclusion criteria. All patients were diagnosed with lung cancer by histopathology or cytology to exclude patients with concurrent primary tumors at other sites. Patients with repeated admissions and no blood type data were excluded. Moreover, the type of lung cancer that was not clear was excluded. A total of 7681 patients with lung cancer were enrolled. The control group was screened for non-lung cancer patients from respiratory and thoracic surgery. A total of 12,671 non-lung cancer patients were included as controls.

### Data collection

The clinical data of the patients were acquired from the hospital information system of the First Affiliated Hospital of Nanchang University, including gender, age, place of residence, smoking history, history of alcohol intake, ABO blood group, and pathological types: squamous carcinoma, adenocarcinoma, small cell lung cancer, large cell lung cancer, and other types. Patients’ routine blood test reports were collected through the laboratory information management system.

### Statistical analysis

SPSS 26.0 statistical software was used for data analysis. The chi-square test was used for inter-group comparison of qualitative data. If the variable missing rate was over 20%, the variable was eliminated. If it was less than 20%, the mean or median was used for filling. The quantitative data followed a normal distribution and were described by mean ± standard deviation (X ± S). The relationship between ABO blood group and different types of lung cancer was analyzed by logistic regression. The figures of the article are drawn by GraphPad Prism 8 software. A two-sided probability value of *P* < 0.05 was a priori taken as significant.

## Results

### Patient characteristics

Table 1 summarizes the clinical characteristics of the subjects enrolled in the present study. The lung cancer group included 7681 patients. Among them, there were 4520 cases of lung adenocarcinoma, 2286 cases of lung squamous cell carcinoma, and 725 cases of small cell carcinoma. In the control group, there were 12,671 patients, including 7986 males and 4685 females, smoking 4270 cases, and drinking 2395 cases. The average age was 58.05 years.

**Table 1:** Baseline characteristics of the study population

Variables	Classes	Control group (***n*** = 12671)	Lung cancer group			
			All (***n*** = 7681)	Lung adenocarcinoma group	Lung squamous cell carcinomas group	Small cell lung cancer group
Gender, *n* (%)	Female	4685 (37.0%)	2545 (33.1%)	2242 (49.6%)	179 (7.8%)	98 (13.5%)
	Male	7986 (63.0%)	5136 (66.8%)	2278 (50.3%)	2107 (92.1%)	627 (86.4%)
Age years, X ± S		58.05 ± 17.58	62.50 ± 10.25	60.63 ± 10.83	65.81 ± 9.25	64.42 ± 9.12
Smoking, *n* (%)	Yes	4270 (33.7%)	3491 (47.1%)	1372 (31.6%)	1570 (71.0%)	455 (64.6%)
	No	8401 (66.3%)	3916 (52.8%)	2976 (68.3%)	640 (28.9%)	249 (35.3%)
Drinking, *n* (%)	Yes	2395 (18.9%)	1780 (24.0%)	830 (19.1%)	709 (32.1%)	201 (28.6%)
	No	10276 (81.1%)	5629 (76.0%)	3520 (80.9%)	1501 (67.9%)	503 (71.3%)

### ABO distribution

ABO blood group distribution of different types of lung cancer and control group is shown in Table 2. We used chi-square analysis to compare all blood types between lung cancer group and control group. The results showed that there were significant differences in ABO blood group composition between lung cancer group and control group (*P* < 0.01). In lung cancer group, blood type B and blood type O were significantly higher than those in control group, while blood type AB was significantly lower than those in control group. Further pairwise comparison showed that there were differences in ABO blood group composition between lung adenocarcinoma, lung squamous carcinoma and small cell lung cancer, and the control group, and the results were statistically significant (*P* < 0.01). Among them, blood type AB in patients with lung adenocarcinoma was less than that in the control group, and blood type O was more than that in the control group. Blood type B and blood type O in patients with lung squamous cell carcinoma were significantly higher than those in the control group, and blood type AB was significantly less than that in the control group. Blood type B blood in patients with small cell lung cancer was significantly higher than that in the control group, and blood type AB was significantly less than that in the control group.

**Table 2:** ABO blood group distribution in control group and different pathological types of lung cancer

Classes	***n***	A	B	AB	O	***p***-value
Control group	12671	4131 (32.6%)	3146 (24.8%)	3634 (28.6%)	1760 (13.8%)	
Lung cancer group	7681	2514 (32.7%)	2028 (26.4%)	1919 (24.9%)	1220 (15.8%)	< 0.01
Lung adenocarcinoma group	4520	1463 (32.3%)	1168 (25.8%)	1165 (25.7%)	724 (16.0%)	< 0.01
Lung squamous cell carcinomas group	2286	759 (33.2%)	612 (26.7%)	555 (24.2%)	360 (15.7%)	< 0.01
Small cell lung cancer group	725	237 (32.6%)	211 (29.1%)	158 (21.7%)	119 (16.4%)	< 0.01

### Logistic regression analysis

**Fig. 1:**
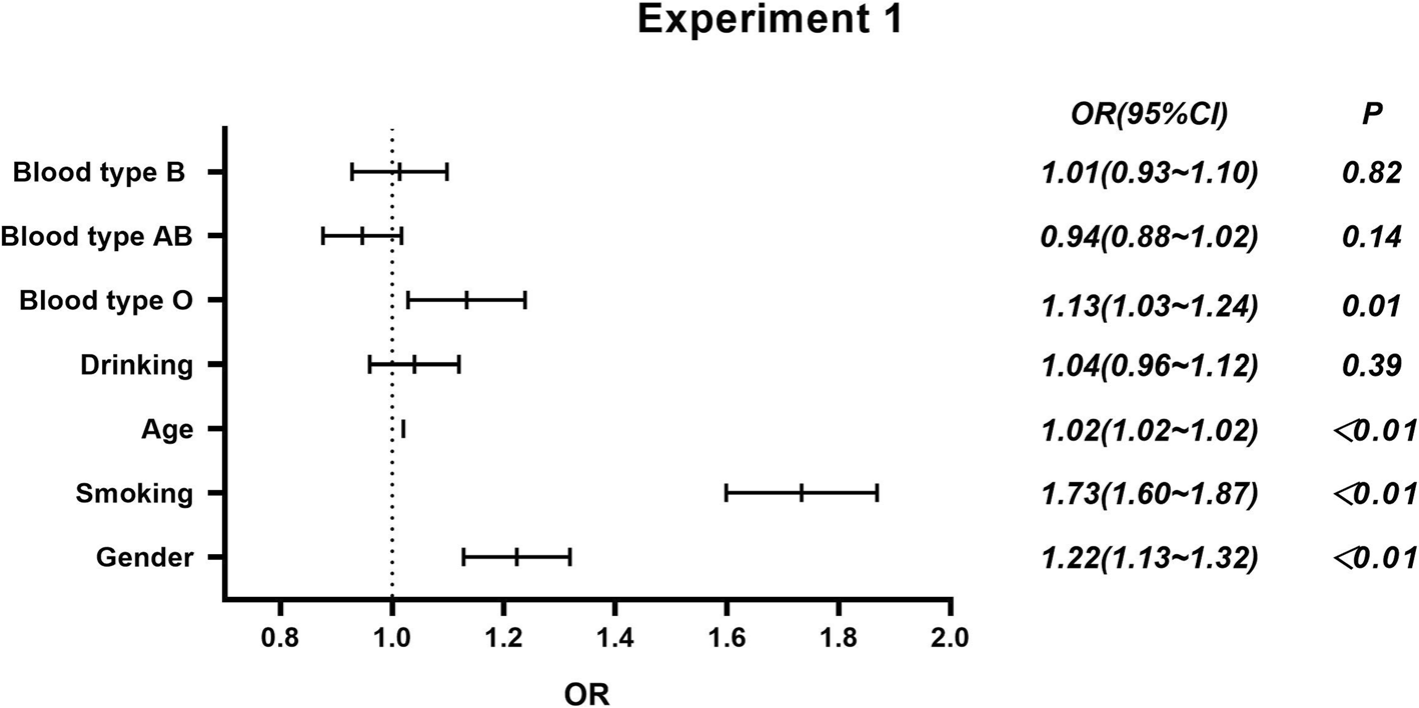
Logistic regression analysis of lung cancer and ABO blood group

**Fig. 2:**
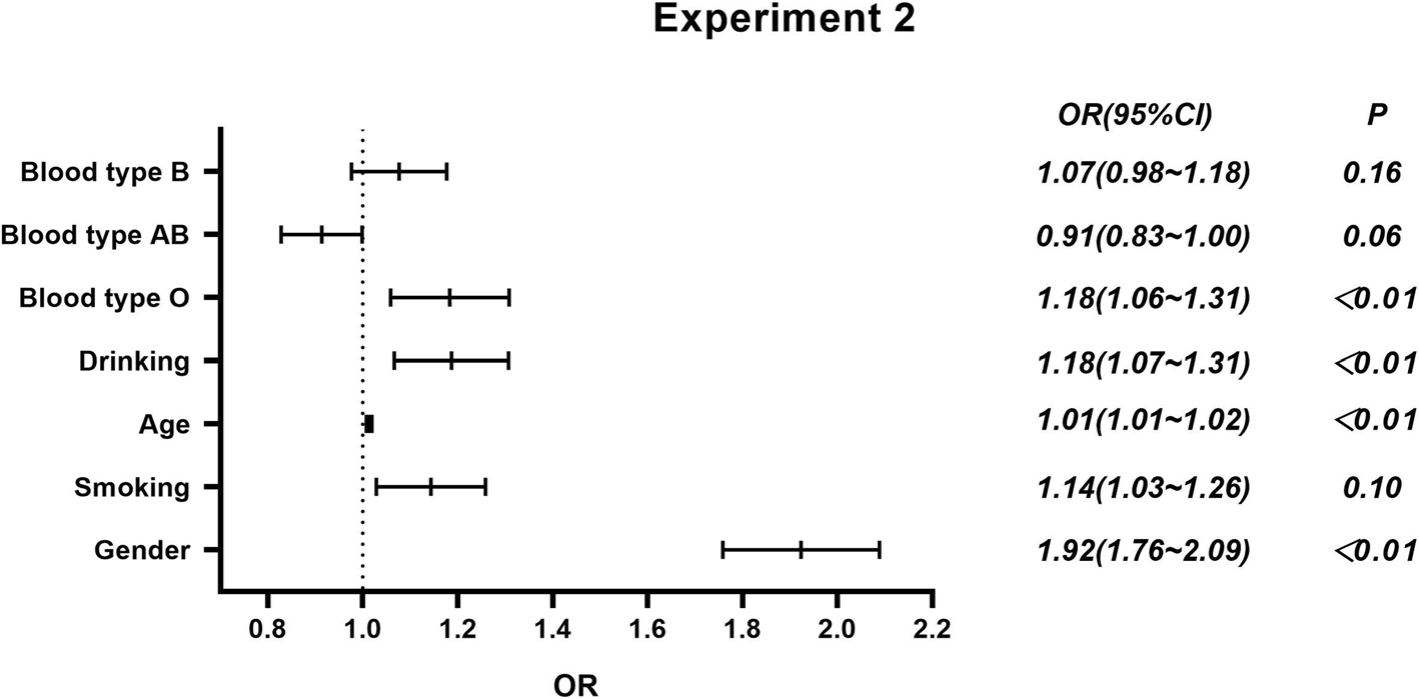
Logistic regression analysis of lung adenocarcinoma and ABO blood group

**Fig. 3:**
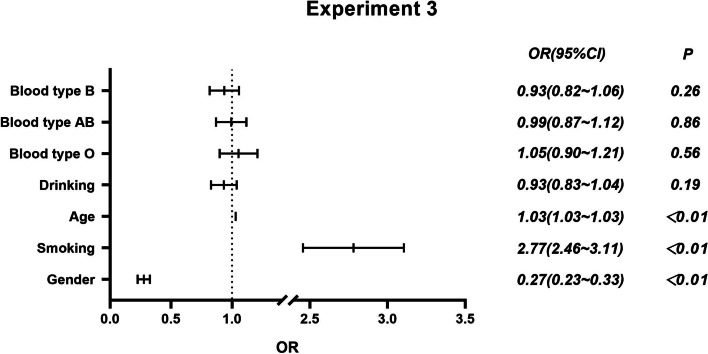
Logistic regression analysis of lung squamous cell carcinomas and ABO blood group

**Fig. 4:**
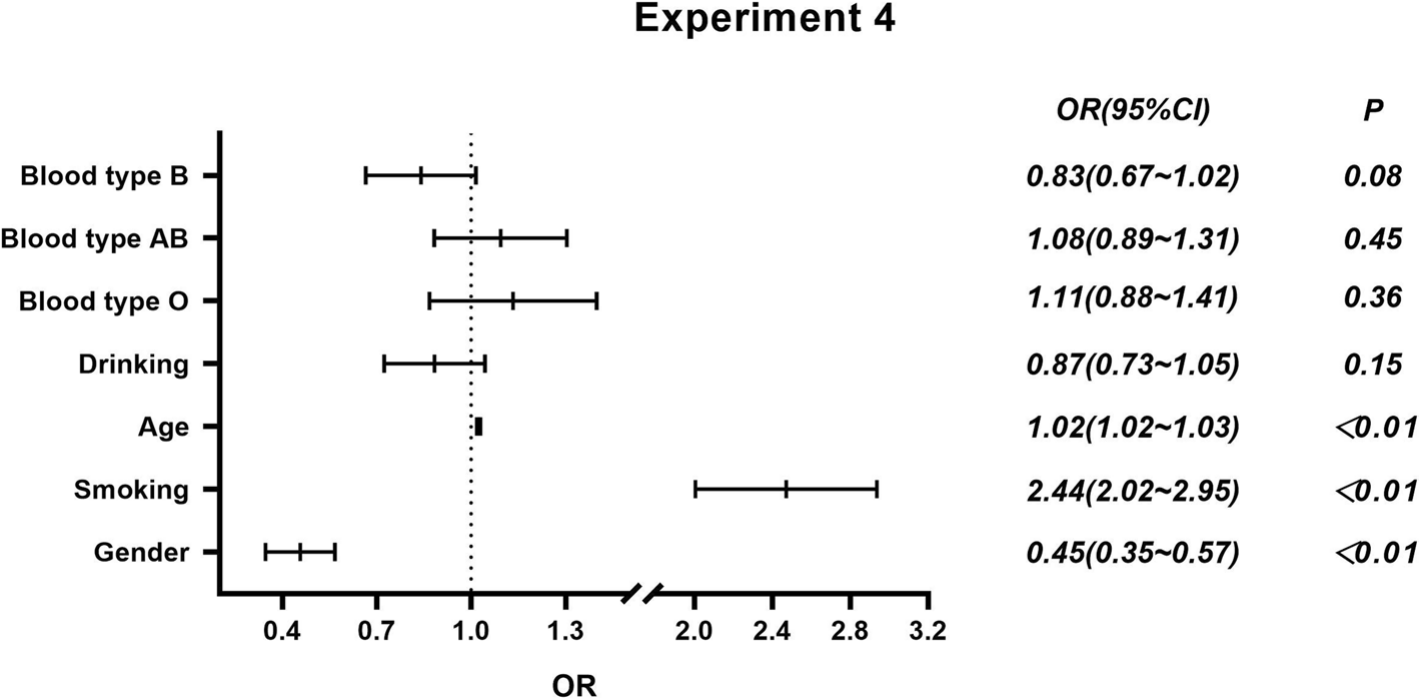
Logistic regression analysis of small cell lung cancer and ABO blood group

Figures 1, 2, 3 and 4 shows the logistic analysis of lung cancer with different pathological types and ABO blood group. Experiment 1 was a logistic analysis between lung cancer group and ABO blood group (Fig. 1). Experiment 2 was a logistic analysis between lung adenocarcinoma group and ABO blood group (Fig. 2). Experiment 3 was a logistic analysis between lung squamous carcinoma group and ABO blood group (Fig. 3). And experiment 4 was a logistic analysis between small cell lung cancer and ABO blood group (Fig. 4). In each group, the control group = 0 and the cancer group = 1 were the dependent variables. Age, gender (male = 0, female = 1), smoking (no = 0, yes = 1), drinking (no = 0, yes = 1), and ABO blood group (*A* = 0, *B* = 1, *AB* = 2, *O* = 3, the odds ratio is calculated based on each blood type against blood type A) were independent variables. Then, the logistic regression analysis was performed.

The results showed that (1) in experiment 1, blood type O had a higher risk of lung cancer than blood type A [*OR* = 1.13 (95% *CI*: 1.03, 1.24)]. Smokers, women, and the elderly are at higher risk of lung cancer (*P* < 0.01). (2) In experiment 2, after adjusting for age, sex, smoking history, and drinking history, blood type O was associated with a higher risk of lung adenocarcinoma than blood type A [*OR* = 1.18 (95% *CI*: 1.06, 1.31)]. In comparison between lung adenocarcinoma and non-lung cancer population, women had a higher risk of lung adenocarcinoma than men [*OR* = 1.92 (95% *CI*: 1.76, 2.09)]. Older people had a higher risk of lung adenocarcinoma [*OR* = 1.01 (95% *CI*: 1.01, 1.02)]. Drinkers had an increased risk of lung adenocarcinoma compared with nondrinkers [*OR* = 1.18 (95% *CI*: 1.07, 1.31)]. (3) In experiment 3, there was no association between blood type and lung squamous cell carcinomas after adjustment for age, sex, smoking, and alcohol consumption. The risk of lung squamous cell carcinomas in women is lower than that in men [*OR* = 0.27 (95% *CI*: 0.23, 0.33)]. Older people had a higher risk of lung squamous cell carcinomas [*OR* = 1.03 (95% *CI*: 1.03, 1.03)]. Smokers had a higher risk of lung squamous cell carcinomas than nonsmokers [*OR* = 2.77 (95% *CI*: 2.46, 3.11)]. In experiment 4, after adjusting for age, sex, smoking, and drinking, there was no significant difference in the risk of small cell lung cancer among different blood types. Women had a lower risk of small cell lung cancer than men [*OR* = 0.45 (95% *CI*: 0.35, 0.57)]. Older people [*OR* = 1.02 (95% *CI*: 1.02, 1.03)] and smokers [*OR* = 2.44 (95% *CI*: 2.02, 2.95)] had a higher risk of small cell lung cancer.

## Discussion

The purpose of this study aims to investigate the influence of the ABO blood group on susceptibility to different pathological types of lung cancer. The results prove that after adjusting for age, sex, smoking, and drinking history, people with blood type O have a higher risk of lung cancer than people with blood type A. When looking into the different types of lung cancer, it turns out that people with blood type O have a higher risk of lung adenocarcinoma than people with blood type A. Furthermore, ABO blood group has no correlation with lung squamous cell carcinoma and small cell lung cancer.

The distribution of ABO blood group in China is characterized by a gradual decrease in the frequency of the B gene and an increase in the frequency of the O gene from north to south, with an increase in the frequency of the A gene in Yunnan, Guizhou, Sichuan, and the middle and lower reaches of the Yangtze. Overall, the blood group distribution in China is O>A>B>AB [9, 10]. However, in this experiment, the blood group distribution of the non-lung cancer control group was A>AB>B>O. This may be due to differences in geographical location and genetic factors. Nevertheless, the patients in the case group and the control group are of the same origin, and the blood group distribution in the case group and the control group largely coincides. Furthermore, the pathophysiological mechanisms between blood group and lung cancer do not change depending on the distribution of blood groups.

This study is not consistent with other relevant studies on the effect of the ABO blood group on the risk of lung cancer. These contradictions may be due to some factors: (1) most of the studies did not distinguish between the pathological types of lung cancer, or only a single pathological type of lung cancer was selected. However, the influence of blood group distribution on the different pathological types of lung cancer cannot be ignored. In view of this, many different pathological types were included in this study, and lung cancer with different pathological types was studied, respectively. What is more, cases with other pathological types were strictly excluded. The results are more reliable. (2) Each study’s sample size differs, and the results may differ. In this study, a larger sample size was adopted to reduce the error caused by the sample size.

The classification of ABO blood groups is determined by the A or B antigens on the surface of red blood cells [11]. People with blood type A have only antigen A on their red blood cells, while people with blood type B have only antigen B on their red blood cells. People with blood type O have neither A nor B antigens in their red blood cells. In contrast, people with blood type AB have both A and B antigens. The gene, 9q34.1–34.2, encodes a blood group antigen-associated glycosyltransferase. However, A and B antigens are not only expressed on the surface of red blood cells but also on epithelial and endothelial cells and in lung tissues [12]. They undertake the functions of cell adhesion, signal transduction, and transport [13]. Studies have found that 9q34 contains proto-oncogene c-abl and human DNA repair gene XPA, and increased tumor susceptibility occurs when the above genes are mutated and lost [14]. In addition, a large prospective cohort study found that increased glycosyltransferase activity corresponding to the ABO allele subtype was associated with an increased risk of pancreatic cancer [15]. On the other hand, the underlying mechanisms associated with ABO blood group and tumorigenesis also include the body’s inflammatory state. ABO blood group has been found to be associated with circulating levels of TNF-α, soluble ICAM-1, e-selectin, and *p*-selectin [16–18]. This suggests that blood type may influence inflammation throughout the body and contribute to the development of cancer.

However, even if a certain genetic characteristic of the organism is susceptible to disease, such susceptibility will not be manifested without the effect of environmental factors [19]. Smoking is a common cause of lung cancer. The Chinese prospective cohort study of chronic diseases carried out by Chen et al. included 500,000 subjects aged 30 to 79 years. The 7-year follow-up showed that the risk of lung cancer in male and female smokers was 2.51 and 2.28 times higher than that in nonsmokers, respectively [20]. Among the specific types of lung cancer, squamous cell lung cancer and small cell lung cancer are greatly affected by smoking [21]. This conclusion is consistent with the results of this study. The occurrence of lung cancer also has a certain relationship with gender. Men are more likely to develop lung squamous cell carcinoma and small cell carcinoma than women, and adenocarcinoma of the lung is less likely than women [22–24]. This conclusion is also consistent with the results of this study. The reason may be that gender is influenced by factors such as smoking and genetics [22]. In addition, lung cancer is an aging disease to some extent. In the cell cycle of continuous replication, telomeres continue to shorten. Then, there is an increasing chance of DNA damage [22]. Therefore, the risk of lung cancer increases gradually with age [25]. The underlying mechanism needs to be further studied.

Shortcomings of this study are as follows:

1. The samples of this study are from a single-center retrospective study. Most of the cases were from Jiangxi province, with particular geographical limitations and single genetic background.
2. Staging analysis of patients with lung cancer was not performed.
3. Confounding factors, such as occupational carcinogenic factors, air pollution, diet, and physical activity, are not included in the study results, which may be biased.

Future multicenter, prospective studies are needed to verify the conclusions of this study.

In conclusion, the ABO blood group has a certain correlation with lung cancer in Jiangxi province. For different pathological types of lung cancer, people with blood type O have a higher risk of lung adenocarcinoma than those with blood type A. Squamous cell lung cancer and small cell lung cancer are not associated with blood type. The findings provide important clues for further research on lung cancer susceptibility.

## Data Availability

Data and material supporting our findings are presented on request.
